# International Society for Computational Biology Honors David Eisenberg with 2013 Accomplishment by a Senior Scientist Award

**DOI:** 10.1371/journal.pcbi.1003116

**Published:** 2013-06-13

**Authors:** Christiana N. Fogg, Diane E. Kovats

**Affiliations:** 1 Freelance Science Writer, Kensington, Maryland, United States of America; 2 International Society for Computational Biology, La Jolla, California, United States of America

## Introduction

The International Society for Computational Biology (ISCB; http://www.iscb.org) honors a senior scientist each year for his or her outstanding achievements. The ISCB Accomplishment by a Senior Scientist recognizes a leading member of the computational biology community for his or her significant contributions to the field through research, service, and training. The 2013 ISCB Accomplishment by a Senior Scientist Award honors Dr. David Eisenberg of the University of California Los Angeles (UCLA).

Dr. Eisenberg (Image 1) was selected the by ISCB's awards committee, which is chaired by Dr. Alfonso Valencia of the Spanish National Cancer Research Center (CNIO) in Madrid. Dr. Eisenberg will receive his awards and deliver a keynote address at the ISCB's 21^st^ annual Intelligent Systems for Molecular Biology (ISMB) meeting. This meeting is being held jointly with the 12^th^ European Conference on Computational Biology and will take place in Berlin, Germany on July 19–23, 2013 (http://www.iscb.org/ismbeccb2013).

**Figure:**
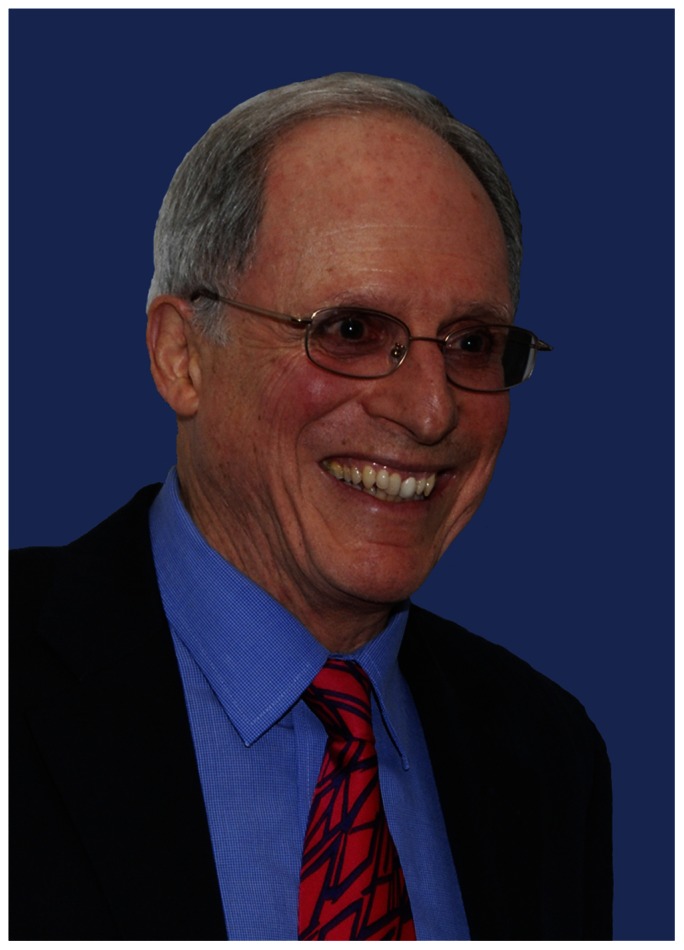
Image 1. David Eisenberg, UCLA. Photo courtesy of Penny Jennings, University of California, Los Angeles.

## 2013 ISCB Accomplishment by a Senior Scientist Award: David Eisenberg

David Eisenberg's love of medicine and science was cultivated first during his childhood by his father, a gentle and beloved pediatrician. Eisenberg recalled, “Every night after dinner he would make house calls. I saw how appreciated—even loved—he was in our village.”

Eisenberg's father also stoked his scientific curiosity by encouraging him to try some experiments in their basement, including attempts to petrify an egg and to grow worms in chocolate. Eisenberg reminisced, “None of these [experiments] worked, but they were fun!”

Eisenberg strongly considered following in his father's footsteps and pursuing a career in medicine. With that goal in mind, he focused his undergraduate studies on biochemical sciences at Harvard University. As a sophomore, he was assigned to Dr. John T. Edsall as a tutor.

Edsall was a pioneering researcher in the field of biophysical chemistry, and under his guidance, Eisenberg had his first encounter with laboratory research. “In my junior year, he assigned me to read scientific papers, most of which baffled me, and at the end of that year, I started a research project in his lab, which became the subject of my senior thesis,” Eisenberg recounted. “After graduation, Dr. Edsall turned my thesis into a short paper which was published in *Science*.”

In spite of Eisenberg's eye-opening undergraduate research experiences, he applied and was accepted to medical school. Edsall was also trained as a medical doctor, but Eisenberg remembered how “Dr. Edsall convinced me that if my goal was to improve the health of mankind, I might have a greater impact working in biochemistry, than as a practicing physician.”

Eisenberg took Edsall's advice to heart and “finessed making an immediate choice by going to Oxford to study theoretical chemistry under Dr. Charles Coulson, one of the founders of quantum chemistry.” Edsall's guidance had also given him a strong foundation in math and physics, which served him well as a graduate student at Oxford as he recalled being “(just) able to work with Coulson on the energetics of hydrogen bonding.”

Eisenberg's postdoctoral studies took him to Princeton in 1964 to work with Dr. Walter Kauzmann, well known for his discovery of the hydrophobic interaction. Eisenberg recollected his ambitious postdoctoral plan “to compute the energy of the hydrophobic interaction in myoglobin, the first protein with a known 3D structure. This plan now seems hopelessly naïve: computers were not yet up to such a calculation, potential functions and theory had not advanced to the point that this was a practical problem, and the early protein crystallographers were not eager to release their atomic coordinates.”

In light of these challenges, Eisenberg's work with Kauzmann culminated in “a monograph on ice and water, which, incidentally, is still in print 44 years later.”

His failed postdoctoral research plan also opened his eyes. He knew that if he wanted to pursue protein energetics, which required knowing protein coordinates, he had to learn X-ray crystallography. Eisenberg's next postdoc took him “to Caltech to study X-ray crystallography with Richard Dickerson, who had been part of the team who had determined the structure of myoglobin.”

His X-ray crystallography training was pivotal to establishing his own lab at UCLA, which focused on studying diverse protein structures. Melittin, a component of bee venom, was one of the first structures he determined with his then graduate student Tom Terwilliger. Eisenberg vividly recalled that, “At last I was able to get down to energetic calculations on a protein, and came up with the idea of the hydrophobic moment. This and related ideas gave me, for the first time, the feeling that I could make discoveries.”

Eisenberg also remembers the excitement of solving the structure of diphtheria toxin dimer, which he worked on with John Collier, Senyon Choe, and Melanie Bennett (Brewer). He recalled the excitement that stemmed from Bennett's observation that “two monomers of the dimer swapped their third domains, and we called this phenomenon “3D domain swapping.” We explored the implications of 3D domain swapping, again calling on my background in energetics. Diphtheria toxin was the first structural example of 3D domain swapping; now there are hundreds.”

Eisenberg's work on protein structures awakened his interest in how protein sequences related to 3D structures. While on sabbatical at the Laboratory of Molecular Biology in Cambridge, he worked with Andrew McLachlan and Mike Gribskov to develop methods to examine protein sequences and use profile analysis to predict the presence of potential structural motifs. These studies led to his work on 3D profiles with Jim Bowie and Roland Luethy, which Eisenberg has now seen “applied to many protein problems.”

Burkhard Rost, president of the ISCB, considers Eisenberg's work on hydrophobicity profiling as groundbreaking because it “describes an important feature of the constituents of proteins (amino acids), namely their preferences to stay away from the solvent water (hydro = water, phobie = animosity). Many other outstanding, original methods followed for the prediction of protein structure and function; many of those methods were so visionary that they started entire fields of research.”

The availability of the first complete genome sequences in the late 1990s inspired Eisenberg's work with “colleague Todd Yeates and our two talented postdoctoral fellows Edward Marcotte and Matteo Pellegrini, [in which] we found we could extract information on protein interactions from sequenced genomes.” These cutting edge studies resulted in several publications that showed how protein function and protein-protein interactions could be predicted from genome sequences.

Eisenberg has focused his research over the last decade on studying amyloid-forming proteins. Several neurodegenerative diseases are associated with amyloid-forming proteins, including Alzheimer's, Parkinson's and amyotrophic lateral sclerosis (Lou Gehrig's) disease. “Just before the turn of the century, I realized that amyloid diseases represent the greatest unmet medical problem facing the world,” Eisenberg recounted. “And at the same time, I realized that structural and computational biology, which have illuminated other areas of biomedicine so well, have not been widely applied to the fundamental problems of amyloid disease. In particular, there had been almost no single crystal X-ray studies of amyloid-forming proteins.”

The use of computational biology with this structural data has helped support the definition of the “amyloid state” of proteins. “Bioinformatics and computational biology are great partners with structural biology. Using the tools together can be surprisingly powerful,” said Eisenberg. Eisenberg's group has studied the structural basis of how normal proteins convert to amyloid fibrils. They have gained great insight into this conversion process by determining the atomic structures of the spines of many different types of amyloid fibrils. Eisenberg also acknowledges that, “Having several friends afflicted with amyloid disorders is a great inspiration. I would love to be able to help them, and others. If we can, it would validate Dr. Edsall's advice that sometimes biochemists can do as much, or more, to help mankind than physicians.”

Eisenberg remains humble about his accomplishments. When asked about being the recipient of the ISCB Senior Scientist Accomplishment Award, he felt “honored, but perhaps over-honored. There are many others who are equally, or more, deserving of this recognition.” But he also recognizes that this award helps highlight the importance of studying amyloid diseases, especially by using the tools of computational biology.

Eisenberg speaks warmly of the mentors that have guided and shaped his scientific training. “I was enormously fortunate to find myself in the research groups of four great mentors: John Edsall, Charles Coulson, Walter Kauzmann, and Richard Dickerson, not to mention my father. All were creative scientists, and also humanists. Watching them I saw their pleasure in scientific discovery, and also saw their insistence on fairness to all those involved in the process of science.”

Their examples have not only served him well as a scientist, but also as a mentor. Eisenberg delights in working with trainees because he loves “their eagerness to learn and to succeed, and their willingness to think freshly about hard problems.”

Eisenberg's scientific curiosity remains insatiable, and when asked for advice to motivate young scientists, his sage answer was “work on fundamental problems, maintain your curiosity, and above all, persevere.”

## Additional Information

Over the past 20 years, the ISMB conference has grown to become the world's largest bioinformatics/computational biology conference, and ISMB/ECCB 2013 will be the year's most important computational biology event globally.

The ISMB conferences provide a multidisciplinary forum for disseminating the latest developments in bioinformatics/computational biology. ISMB/ECCB brings together scientists from computer science, molecular biology, mathematics, statistics, and related fields. Its principal focus is on the development and application of advanced computational methods for biological problems. ISMB/ECCB 2013 offers a strong scientific program and the broadest scope of any international bioinformatics/computational biology conference.

ISMB/ECCB 2013 takes place July 19–23, at the Messe Berlin (ICC Berlin), Germany. For two days preceding the conference, additional opportunities including Satellite Meetings, Junior Principal Investigator Symposium, Student Council Symposium, and a selection of Special Interest Group Meetings and Tutorials are all offered to enable registered participants to learn more on the latest methods and tools within specialty research areas.

